# IDENTIFYING CHALLENGES FOR AGE-INCLUSIVE HIGHER EDUCATION: SURVEY RESPONSES BY FACULTY, STAFF, AND STUDENTS

**DOI:** 10.1093/geroni/igad104.0641

**Published:** 2023-12-21

**Authors:** Lauren Bowen

**Affiliations:** University of Massachusetts Boston, Boston, Massachusetts, United States

## Abstract

To ground and contextualize focus group discussions, potential challenges to age-inclusivity were identified from open-ended responses in a survey of faculty, staff, and students at 25 AFU institutions in the United States. First-level coding identified 2,368 responses that indicated participants’ perceptions of their campus’s efforts to be age-friendly across major function areas of higher education institutions; of these, many responses directly addressed key functions that the current study aims to address: 508 on Teaching and Learning; 260 on Student Affairs; 348 on Personnel. Second-level coding identified particular themes capturing common negative perceptions by faculty, staff, and students within each function area. For example, in teaching and learning, the four common complaints were that classroom practices and environments were not age-inclusive; that accessing appropriate academic support outside the classroom was difficult for older/nontraditional students; and that classes could do more to maximize the benefits of age diversity. As potential challenges to age-inclusivity in higher education, these themes provided the base structure of 8 focus group discussion guides tailored to function area and constituent group: Teaching and Learning (for faculty, staff, and students), Student Affairs (for faculty, staff, and students), and Personnel (for faculty and staff).

